# Number of failed organs and response to therapy determine outcome in patients with acute pancreatitis requiring level 1 organ support

**DOI:** 10.1186/cc10994

**Published:** 2012-03-20

**Authors:** G Morris-Stiff, A Baker, A Breen, A Smith

**Affiliations:** 1 Leeds Teaching Hospitals, Leeds, UK

## Introduction

The aim of the study was to establish if the number of organs failing at admission to the ICU and the response to support had a bearing on outcome in patients with severe acute pancreatitis (SAP).

## Methods

Only SAP patients requiring organ support were included in the analysis. Gallstones (55%) and alcohol were the commonest aetiologies. The proportion of patients with one, two or three system failures at baseline, 24, 48, and 72 hours were calculated and related to outcome.

## Results

A total of 123 patients (85 male and 38 female) with a mean age of 58 years met the study criteria. The numbers of patients presenting with one, two and three organ failures were 29, 70 and 24 respectively, of which the mortality was six (21%), 29 (41%) and 14 (48%). Subsequent figures were 24, 57 and 39 with mortalities of four (17%), 19 (33%), and 24 (62%) at 24 hours; 21, 53 and 43 with mortalities of two (10%), 18 (34%), and 26 (60%) at 48 hours; and 17, 49 and 45 with mortalities of zero (0%), 16 (33%), and 28 (62%) at 72 hours.

## Conclusion

These data allow prognostication of patients with SAP requiring organ support. At 72 hours, the prognosis of patients with single organ failure is excellent and that of patients with three-organ failure remains poor.

